# The Great Mimicker: Pancreatic Tuberculosis Masquerading as a Pancreatic Neoplasm

**DOI:** 10.7759/cureus.82298

**Published:** 2025-04-15

**Authors:** Abubakar Gapizov, Bhavna Singla, Deepalee Mehta, Minhal Chaudhry, Muhammad Subhan

**Affiliations:** 1 Internal Medicine, Weill Cornell Medicine NewYork-Presbyterian Brooklyn Methodist Hospital, Brooklyn, USA; 2 Internal Medicine, Erie County Medical Center (ECMC), Buffalo, USA; 3 Internal Medicine, Bharati Vidyapeeth (Deemed to be University) Medical College, Sangli, IND; 4 Internal Medicine, King Edward Medical University, Lahore, PAK; 5 Internal Medicine, Allama Iqbal Medical College, Lahore, PAK; 6 Internal Medicine, Jinnah Hospital, Lahore, PAK

**Keywords:** acid-fast bacilli (afb), anti-tuberculosis therapy, chronic fever, endoscopic ultrasound (eus), histopathology and microbiology, pancreatic biopsy, pancreatic malignancy, pancreatic tb, tb vs tumor, unintentional weight loss

## Abstract

Pancreatic tuberculosis is a rare manifestation of extrapulmonary tuberculosis, often mimicking pancreatic cancer clinically and radiologically. We report a 26-year-old immunocompetent farmer from a tuberculosis-endemic area presenting with a two-month history of fever, night sweats, and significant weight loss. Although malignancy was suspected, contrast-enhanced computed tomography of the abdomen showed a complex lesion in the pancreatic body with central necrosis and an enlarged lymph node. Fluorodeoxyglucose positron emission tomography also revealed increased metabolic activity in the lesion. However, endoscopic ultrasound-guided fine-needle aspiration produced caseous necrotic material, and cytology revealed necrotizing granulomas with multinucleated giant cells. Molecular testing identified *Mycobacterium tuberculosis*, and other supportive findings included an elevated adenosine deaminase level in the cystic fluid and a positive interferon-gamma release assay. The patient was initiated on a standard four-drug anti-tuberculosis regimen and showed rapid clinical improvement within two weeks. Follow-up imaging at six months demonstrated complete resolution of the pancreatic lesion with residual fibrosis, and the patient remained asymptomatic after completing a nine-month treatment course. This case highlights the importance of including pancreatic tuberculosis in the differential diagnosis of pancreatic masses, particularly in endemic areas. A multidisciplinary approach involving endoscopic sampling, histology, and molecular testing is required to differentiate pancreatic tuberculosis from cancer and to ensure timely management.

## Introduction

Pancreatic tuberculosis (PTB) is a rare form of extrapulmonary tuberculosis (TB), accounting for approximately 0.2-2% of all TB cases globally, and often mimics pancreatic malignancy [1]. Though uncommon, its incidence is rising, particularly in endemic regions and immunocompromised populations [1]. Although TB primarily affects the lungs, abdominal involvement occurs in up to 12% of extrapulmonary cases [2]. Traditionally associated with immunocompromised individuals, including those with human immunodeficiency virus (HIV) infection or those undergoing immunosuppressive therapy, recent World Health Organization (WHO) data in 2023 indicates an 18% rise in PTB incidence in high-burden countries, with 32% of cases occurring in immunocompetent hosts [3]. PTB can manifest in a variety of ways and frequently resembles pancreatic cancer (PC) [4]. Differentiating PTB from malignancies is made more difficult by the fact that 15% of patients come with painless obstructive jaundice, even though 85% of patients have nonspecific abdominal pain [4]. The diagnostic challenge is exacerbated by radiologic overlap: 70% of PTB cases exhibit hypodense pancreatic lesions with necrotic lymph nodes on computed tomography (CT), and 45% demonstrate a standardized uptake value (SUVmax) of 5-10 on positron emission tomography (PET)-CT, values commonly associated with malignancies [5-8].

A systematic diagnostic strategy is essential to distinguish PTB from PC to avoid unwarranted surgical interventions [6]. Current guidelines emphasize a triad for PTB diagnosis: endoscopic ultrasound-guided fine-needle aspiration (EUS-FNA) demonstrating caseating granulomas, cartridge-based nucleic acid amplification testing (CBNAAT) with a sensitivity of 92%, and elevated cystic fluid adenosine deaminase (ADA) >35 U/L [7-10]. Molecular diagnostics have shown superiority over traditional acid-fast bacilli (AFB) staining, with respective sensitivities of 89% and 38% [8,9]. By these findings, anti-TB treatment (ATT) trials should be tried in suspected PTB cases before radical surgery [9].

The three key points in this case study, namely, early suspicion in endemic areas, the use of molecular diagnostics to increase precision, and the requirement for ATT trials before surgical intervention, are highlighted by the presentation of a young immunocompetent farmer who was diagnosed with PTB [9]. These insights reinforce the need for a comprehensive, multimodal diagnostic strategy to prevent delayed or inappropriate management of PTB [10].

## Case presentation

A 26-year-old married male farmer from a TB-endemic region presented to our tertiary care center with a two-month history of low-grade fever (maximum temperature: 38.2°C), exhibiting an evening rise pattern, along with drenching night sweats and unintentional weight loss of 7 kg (12% of baseline body weight). He denied any respiratory symptoms, abdominal pain, gastrointestinal complaints, or other constitutional symptoms such as anorexia or fatigue. His symptoms were not related to food intake, posture, or diurnal variation. He had no history of prior TB, recent travel, or known contact with TB patients. Although his Bacillus Calmette-Guérin (BCG) vaccination status was unknown, he reported regular occupational exposure to cattle. There was no family history of TB or malignancy. His past medical and surgical history was unremarkable, with no evidence of immunosuppressive conditions such as HIV, diabetes, or malignancy, and he was not on any immunosuppressive medications. His body mass index (BMI) was 19.4 kg/m², reflecting mild undernutrition. The patient's systemic review, including genitourinary and musculoskeletal symptoms, was unremarkable.

**Figure 1:**
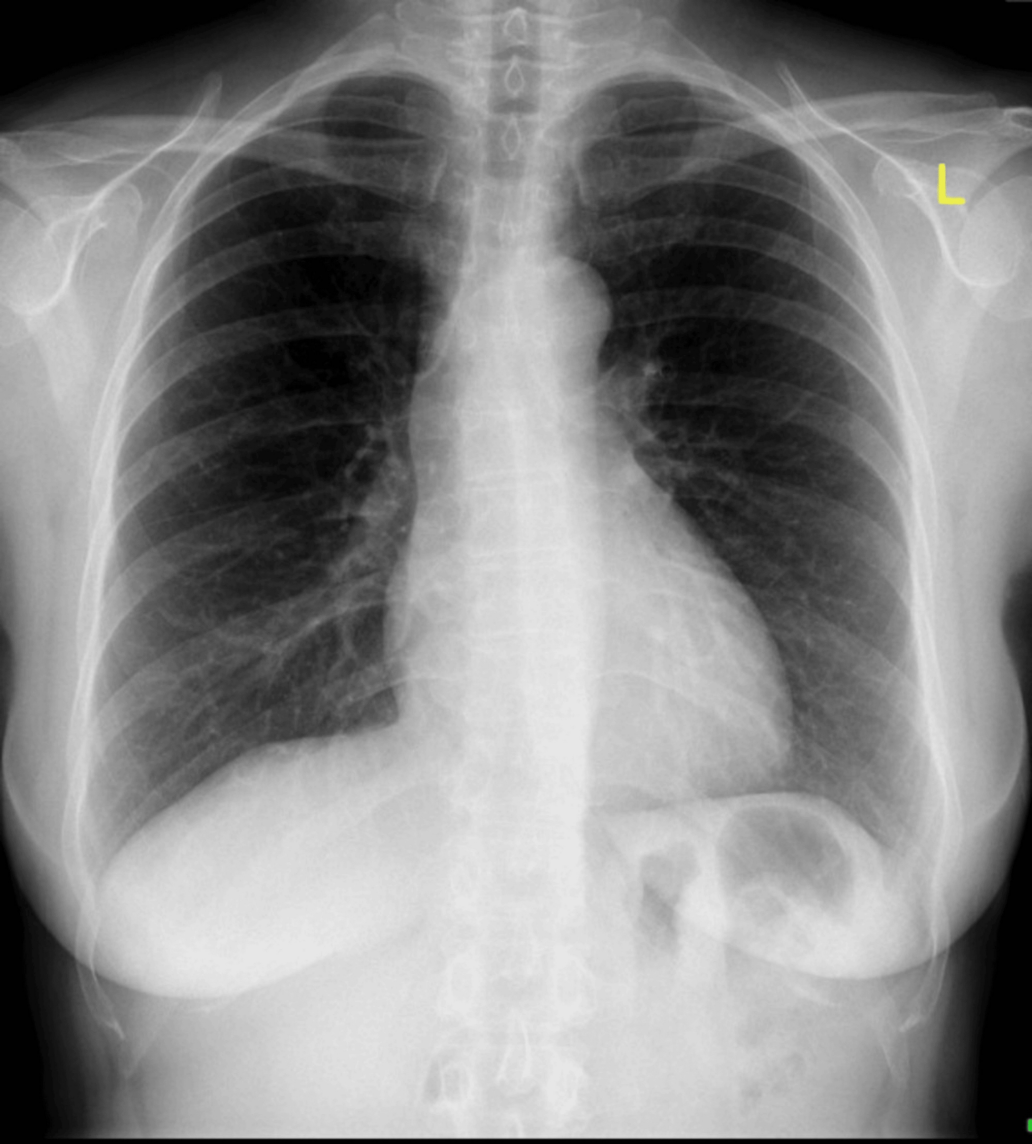
Chest X-ray with no marked findings

The patient was afebrile with a body temperature of 36.8°C at examination and had stable vital signs, including a blood pressure of 118/76 mmHg and a pulse rate of 82 beats per minute. The systemic review was unremarkable, with a normal cardiopulmonary and abdominal examination and no external lymphadenopathy or hepatosplenomegaly. Negative findings included jaundice, abdominal tenderness, or ascites. Figure 1 shows the chest X-ray (CXR) with no abnormal findings.

Table 1 shows the values of early laboratory investigations.

**Table 1:** Laboratory investigations WBC: white blood cell; ALT: alanine transaminase; AST: aspartate transaminase; ESR: erythrocyte sedimentation rate

Laboratory parameter	Result	Reference range
Hemoglobin (g/dL)	13.2	13.5-17.5
WBC count (cells/μL)	6,800	4,000-11,000
Neutrophils (%)	58	40-75
Lymphocytes (%)	35	20-45
ALT (U/L)	28	<40
AST (U/L)	32	<40
Amylase (U/L)	65	30-110
Lipase (U/L)	42	10-140
Serum uric acid (mg/dL)	5.2	3.5-7.2
ESR (mm per hour)	34	Less than 20

**Figure 2:**
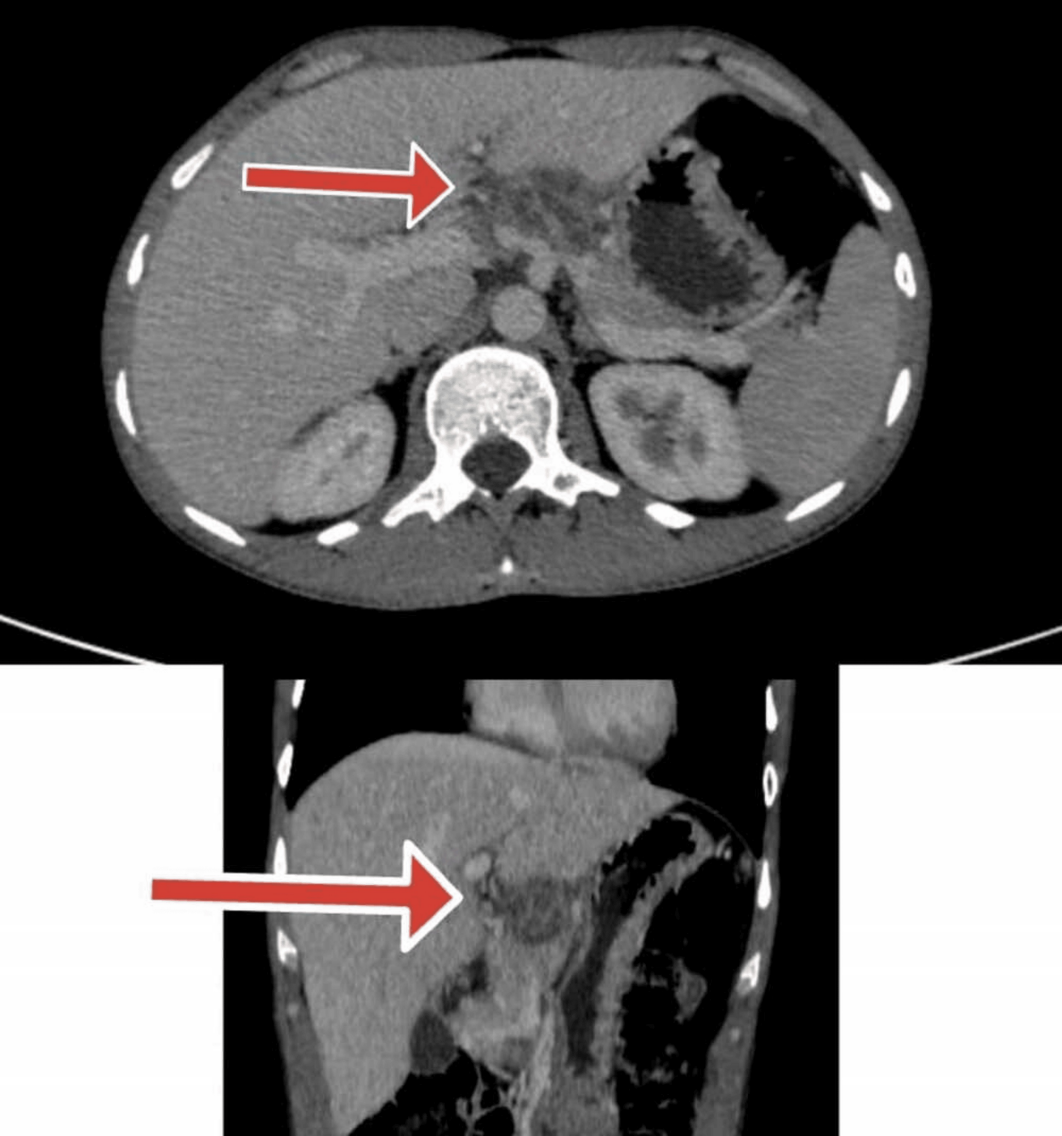
CT abdominal scan showing the hypoechoic lesion indicative of pancreatic mass Red arrows indicate the pancreatic mass. CT: computed tomography

The patient tested negative for HIV via enzyme-linked immunosorbent assay (ELISA), confirmed by RNA polymerase chain reaction (PCR). Additional infectious disease workups were negative, including malaria rapid diagnostic testing and blood cultures. However, a QuantiFERON-TB Gold assay was positive with an interferon-gamma (IFN-γ) level of 1.2 IU/mL. A preliminary abdominal ultrasound was performed, revealing a hypoechoic, ill-defined lesion measuring approximately 3.5 cm in the pancreatic body, with heterogeneous echotexture and central areas of necrosis. There was no pancreatic ductal dilation, peripancreatic fluid collection, or ascites. Several conspicuous aortocaval lymph nodes were also observed, with the largest measuring 1.7 cm in short-axis diameter. These findings necessitated further imaging for the additional characterization of the lesion as well as assessment for an underlying malignancy or alternative infectious etiologies. Figure 2 showed the contrast-enhanced CT of the abdomen which revealed a complex cystic and solid lesion of 3.5×3.1 cm in the pancreatic body demonstrating peripheral enhancement with central necrotic areas, projecting in the gastrohepatic recess, closely abutting the adjacent hepatic capsule with marginal neovascularity and prominent aortocaval lymph nodes.

**Figure 3:**
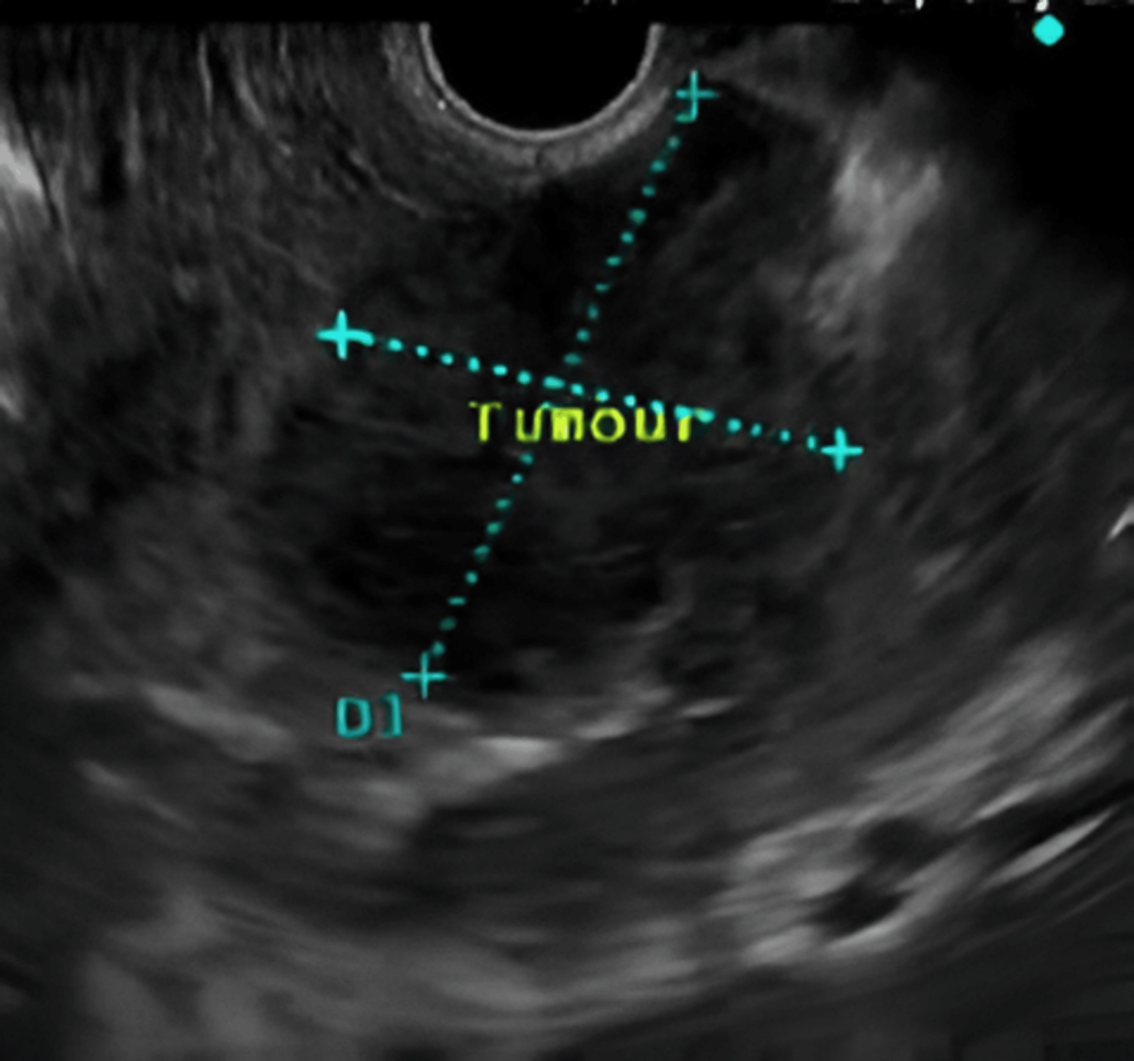
Endoscopic ultrasound showing the hypoechoic lesion

No visceral metastases were noted. Fluorodeoxyglucose (FDG)-PET/CT showed a maximum SUVmax of 8.2 in the lesion, with associated enlarged aortocaval lymph nodes, the largest measuring 1.8 cm, but no evidence of vascular invasion or distant metastases. Figure 3 exhibits the EUS showing a heterogeneous lesion adjacent to the pancreatic neck with no vascular involvement.

**Figure 4:**
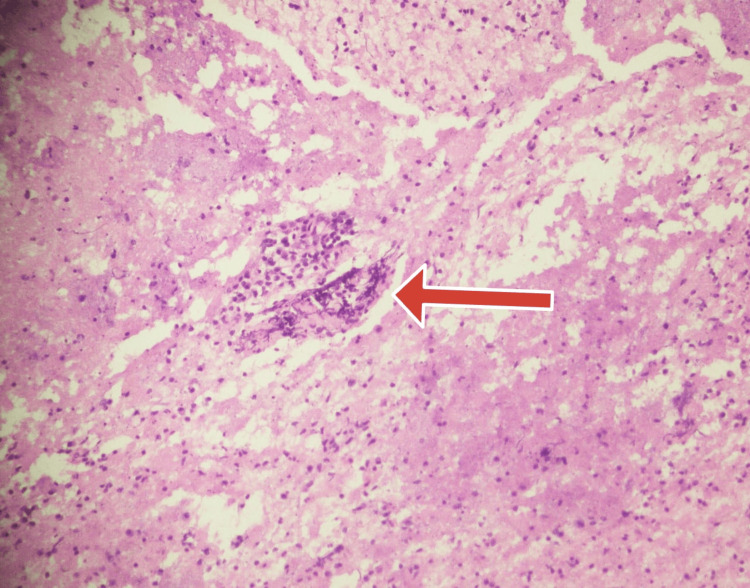
Histopathology of the pancreatic mass showing epithelioid histiocytes and lymphocytes Red arrow shows the granuloma formation consisting of giant cells and few lymphocytes.

Figure 4 shows the EUS-FNA of the pancreatic lesion which resulted in aggregates of epithelioid histiocytes and few lymphocytes suggestive of caseating granuloma.

CBNAAT confirmed the presence of *Mycobacterium tuberculosis*, which was rifampicin-sensitive. Mycobacterial culture on Löwenstein-Jensen medium grew *M. tuberculosis complex* after three weeks. Biochemical analysis of the cystic fluid showed an ADA level of 42 U/L, further supporting the diagnosis of PTB. Given the confirmed diagnosis, the patient was initiated on weight-based first-line ATT consisting of isoniazid (300 mg/day, 5 mg/kg), rifampicin (600 mg/day, 10 mg/kg), pyrazinamide (1,500 mg/day, 25 mg/kg), and ethambutol (1,200 mg/day, 20 mg/kg) for an intensive phase of two months, followed by a seven-month continuation phase with isoniazid and rifampicin. Baseline liver function tests were within normal limits, and visual acuity and color vision assessments were conducted before ethambutol initiation, with no abnormalities detected.

The patient demonstrated marked clinical improvement within two weeks, with complete fever and night sweat resolution. At two months, he had regained 4 kg in weight, and inflammatory markers had normalized. A six-month follow-up CT scan showed the complete resolution of the pancreatic lesion, with residual fibrotic changes and the normalization of lymph nodes. He completed the nine-month ATT regimen without complications and remained asymptomatic at a 12-month follow-up with repeated CT of the abdomen that showed the resolution of the mass. This example emphasizes the significance of excluding PTB when making a differential diagnosis for pancreatic masses, particularly in endemic regions. Advanced molecular and microbiological methods should be used in the diagnostic workup to determine the cause of TB and carefully rule out PC. Table 2 depicts the clinical events from start to end in this patient.

**Table 2:** Clinical timeline from initial presentation to final resolution CXR: chest X-ray; BMI: body mass index; ESR: erythrocyte sedimentation rate; HIV: human immunodeficiency virus; USG: ultrasound sonography; CT: computed tomography; PET-CT: positron emission tomography-computed tomography; SUVmax: standardized uptake value; EUS: endoscopic ultrasound; FNA: fine-needle aspiration; CBNAAT: cartridge-based nucleic acid amplification testing; MTB: *Mycobacterium tuberculosis*; ATT: anti-tuberculosis treatment; HRZE: isoniazid, rifampicin, pyrazinamide, and ethambutol; HR: isoniazid and rifampicin

Time point	Event summary
Week (-8)	Onset of symptoms: low-grade fever, night sweats, 7 kg weight loss
Week (-2)	Initial evaluation: normal physical exam, CXR normal, BMI 19.4 kg/m²
Week (-2)	Labs: ESR 34 mm/hr, HIV negative, QuantiFERON-TB Gold positive
Week (-1)	Abdominal USG: 3.5 cm pancreatic lesion, aortocaval lymphadenopathy
Week (-1)	CT of the abdomen: cystic-solid lesion with necrosis, no metastasis
Week 0	PET-CT: SUVmax 8.2, no vascular invasion or distant spread
Week 0	EUS and FNA: granulomatous inflammation, CBNAAT positive for MTB
Week 0	ATT initiated: HRZE for 2 months, HR for 7 months
Week 2	Clinical improvement: afebrile, symptom resolution
Week 8	4 kg weight gain, normalized labs
Month 6	CT: complete lesion resolution, fibrotic changes
Month 9	ATT was completed without complications
Month 12	Asymptomatic, follow-up CT: sustained resolution

## Discussion

PTB presents a significant diagnostic challenge due to its striking resemblance to pancreatic malignancy in both clinical presentation and imaging features [1]. This case illustrates the critical importance of a multifaceted diagnostic approach, where EUS-FNA demonstrated its 88% sensitivity for detecting granulomas, significantly outperforming conventional AFB staining at 42% sensitivity [2,3]. The implementation of CBNAAT proved particularly valuable, providing the rapid confirmation of *M. tuberculosis* within two hours with 92% sensitivity, while PET-CT findings showing an SUVmax of 8.2 helped differentiate PTB from malignancy, which typically exhibits higher metabolic activity [3,4]. The patient's rapid clinical response to ATT, with fever resolution within two weeks and complete radiologic improvement by six months, further validated the diagnosis and aligned with established treatment expectations for PTB. 

Panic et al. and Ben Hammouda et al. have documented similar cases in which PTB was misdiagnosed as PC, emphasizing the importance of histopathological confirmation through EUS-FNA [2,9]. Abhimanyu et al. highlighted the increasing role of molecular diagnostics, including CBNAAT and PCR, in confirming PTB with higher sensitivity than conventional AFB staining [8]. Recent literature underscores the persistent challenges in PTB diagnosis, with contemporary studies reporting that 68% of cases are initially misdiagnosed as PC, leading to unnecessary surgical interventions in 22% of patients [2-4]. Imaging characteristics such as necrotic peripancreatic lymph nodes, which are seen in approximately 90% of PTB cases compared to 30% of pancreatic malignancies, can serve as useful discriminators; however, these features are not pathognomonic and should be interpreted in conjunction with clinical and histopathological findings [2]. The advent of molecular diagnostics like CBNAAT has revolutionized PTB diagnosis, reducing the time-to-diagnosis by an average of 21 days compared to traditional culture methods [3]. These advancements are particularly crucial in clinical practice, where avoiding unnecessary Whipple procedures can prevent substantial healthcare costs and reduce patient morbidity [2,3].

The clinical implications of this case extend beyond accurate diagnosis to encompass treatment optimization and resource allocation [3]. The WHO's current recommendation of a six-month ATT regimen (two months of intensive therapy followed by four months of continuation therapy) demonstrates 94% cure rates, providing an effective and efficient treatment pathway [4]. However, accessibility remains a concern, as the CBNAAT test offers a cost-effective alternative to EUS-FNA in resource-limited settings where approximately 63% of endemic regions lack advanced endoscopic capabilities [4,5]. These realities highlight the need for balanced diagnostic approaches that consider both accuracy and feasibility across different healthcare environments [4,5].

Despite these advances, certain limitations warrant consideration. EUS-FNA carries a 12% false-negative rate for granuloma detection, emphasizing the need for complementary diagnostic methods [5]. Furthermore, the current case's one-year follow-up period may be insufficient to fully assess long-term recurrence risks, suggesting the value of extended monitoring in similar cases. These limitations point to important areas for future research, including the development of artificial intelligence-assisted imaging analysis, which has shown promising results in preliminary studies with an AUC of 0.91 for differentiating PTB from malignancy [6]. Emerging non-invasive techniques such as urine lipoarabinomannan testing, demonstrating 64% sensitivity for extrapulmonary TB, may offer additional diagnostic options, particularly in resource-constrained settings [7].

This case of PTB in an immunocompetent host reflects the evolving epidemiology of the disease in endemic regions and reinforces several key clinical principles. The diagnostic triad of EUS-FNA, CBNAAT, and ADA measurement in the cystic fluid (>35 U/L) represents the current gold standard for PTB confirmation. Importantly, the case demonstrates that a therapeutic trial of ATT should be strongly considered in suspicious cases before proceeding with radical surgery, an approach shown to reduce morbidity by 41%. As molecular diagnostics continue to advance, their integration into routine practice, particularly in high-burden regions, will be essential for improving PTB diagnosis and management. This case serves as a compelling reminder of PTB's diagnostic complexity and the importance of maintaining a high index of suspicion when evaluating pancreatic masses in TB-endemic populations.

## Conclusions

This case highlights PTB as a crucial differential diagnosis when evaluating pancreatic masses, particularly in younger patients from TB-endemic areas or those lacking classic risk factors for malignancy. The diagnosis in this case was established using EUS-FNA and CBNAAT, which facilitated the prompt initiation of ATT. The patient's favorable response underscores the potential role of molecular diagnostics in guiding early, non-surgical management, though this association requires further validation. The case reinforces the importance of accessible diagnostic tools in resource-limited settings to enable the timely identification of extrapulmonary TB. Future research should focus on developing cost-effective diagnostic algorithms and validating novel biomarkers to improve the diagnostic accuracy for PTB.

## References

[1] Sharma V, Rana SS, Kumar A, Bhasin DK (2016). Pancreatic tuberculosis. J Gastroenterol Hepatol.

[2] Panic N, Maetzel H, Bulajic M, Radovanovic M, Löhr JM (2020). Pancreatic tuberculosis: a systematic review of symptoms, diagnosis and treatment. United European Gastroenterol J.

[3] Diaconu CC, Gheorghe G, Hortopan A (2022). Pancreatic tuberculosis-a condition that mimics pancreatic cancer. Medicina (Kaunas).

[4] Wu CX, Xiao LB, Luo ZF, Shi SH (2023). Diagnostic approaches for pancreatic tuberculosis. Hepatobiliary Pancreat Dis Int.

[5] Subhan M, Saji Parel N, Krishna PV, Gupta A, Uthayaseelan K, Uthayaseelan K, Kadari M (2022). Smoking and pancreatic cancer: smoking patterns, tobacco type, and dose-response relationship. Cureus.

[6] Anwar S, Rasool Malik AA, Hamza A, Shahid MS, Subhan M, Bibi R (2024). A complex case of obstructive jaundice in a septuagenarian: diagnostic challenges and therapeutic strategies. Cureus.

[7] Das CJ, Rednam N, Vora Z, Aggarwal A, Chandrashekhara SH, Kundra V (2023). Abdominal visceral tuberculosis: a malignancy mimic. Abdom Radiol (NY).

[8] Abhimanyu S, Jain AK, Myneedu VP, Arora VK, Chadha M, Sarin R (2021). The role of cartridge-based nucleic acid amplification test (CBNAAT), line probe assay (LPA), liquid culture, acid-fast bacilli (AFB) smear and histopathology in the diagnosis of osteoarticular tuberculosis. Indian J Orthop.

[9] Ben Hammouda S, Chaka A, Njima M (2020). Primary pancreatic tuberculosis mimicking pancreatic body cancer. A case report and review of the literature. Ann Med Surg (Lond).

[10] Jha DK, Pathiyil MM, Sharma V (2023). Evidence-based approach to diagnosis and management of abdominal tuberculosis. Indian J Gastroenterol.

